# Probiotic Species in the Management of Periodontal Diseases: An Overview

**DOI:** 10.3389/fcimb.2022.806463

**Published:** 2022-03-25

**Authors:** Yuwei Zhang, Yi Ding, Qiang Guo

**Affiliations:** ^1^State Key Laboratory of Oral Diseases, National Clinical Research Center for Oral Diseases, Department of Periodontics, West China Hospital of Stomatology, Sichuan University, Chengdu, China; ^2^State Key Laboratory of Oral Diseases, National Clinical Research Center for Oral Diseases, West China Hospital of Stomatology, Sichuan University, Chengdu, China

**Keywords:** probiotic, periodontal disease, periodontopathogen, microecological balance, immunoregulation

## Abstract

Periodontal diseases are one of the most common chronic inflammatory diseases of the oral cavity, which are initiated and sustained by pathogenic plaque biofilms. Central to modern periodontology is the idea that dysbiosis of periodontal microecology and disorder of host inflammatory response gives rise to degradation of periodontal tissues together, which eventually leads to tooth loss, seriously affecting the life quality of patients. Probiotics were originally used to treat intestinal diseases, while in recent years, extensive studies have been exploring the utilization of probiotics in oral disease treatment and oral healthcare. Probiotic bacteria derived from the genera *Lactobacillus*, *Bifidobacterium*, *Streptococcus*, and *Weissella* are found to play an effective role in the prevention and treatment of periodontal diseases *via* regulating periodontal microbiota or host immune responses. Here, we review the research status of periodontal health-promoting probiotic species and their regulatory effects. The current issues on the effectiveness and safety of probiotics in the management of periodontal diseases are also discussed at last. Taken together, the use of probiotics is a promising approach to prevent and treat periodontal diseases. Nevertheless, their practical use for periodontal health needs further research and exploration.

## Probiotic and Oral Health

The term “probiotic” was put forward by Lilly and Stillwell in 1965, defined as “growth-promoting factors produced by microorganisms” (Lilly and Stillwell, 1965). Since then, the definition of “probiotic” has changed several times until the WHO and the Food and Agriculture Organization of the United States (FAO) in 2002 came up with a new definition that was generally accepted: probiotics are “living microorganisms that can have a beneficial effect on the host when taken in sufficient doses” (Hill et al., 2014). The origins of probiotics could be traced back to ancient Roman records, and Plinius Secundus Maior recorded that fermented milk products are beneficial to stomach healing. In the early 20th century, Elie Metchnikoff, a Nobel laureate, recorded in “The Prolongation of Life” that Bulgarians lived longer than others because they drank fermented milk (Metchnikoff, 1907). Through the study of human gut flora, he concluded that harmful products of some bacteria could be a reason for aging, and he recommended milk fermented by *Lactobacillus* to prevent the harmful effects of bacterial products.

Probiotics were originally used to treat intestinal diseases. Studies have shown that they could help control intestinal infections, relieve constipation and diarrhea, improve lactose intolerance, etc. (Lourenshattingh and Viljoen, 2001). The beneficial effects of probiotics defined by WHO and FAO are not only on the intestines but also on other body systems. In fact, many probiotics have been demonstrated to play a role in maintaining a healthy urogenital system and fighting against cancers, diabetes, obesity, and allergies (Waigankar and Patel, 2011; Sunita et al., 2012; Kang et al., 2013; Takeda et al., 2014; Kahouli et al., 2015). In recent decades, extensive studies also explored the application of probiotics in oral disease treatment and oral healthcare. Currently, it is found that probiotics contributing to oral health are concentrated in the genera *Lactobacillus*, *Bifidobacterium*, *Streptococcus*, and *Weissella*, as well as certain scattered species like *Bacillus subtilis* and *Saccharomyces cerevisiae.* Several strains of *Lactobacillus reuteri*, *Lactobacillus brevis*, *Streptococcus salivarius*, etc., have been commercially produced as oral health-promoting probiotics, all of which are microorganisms isolated from the oral cavity (Allaker and Stephen, 2017; Mahasneh and Mahasneh, 2017). Effects of probiotics on improving oral health have been observed in common oral diseases such as dental caries, periodontal diseases, oral candida infection, and halitosis (Ince et al., 2015; Ohshima et al., 2016; Yoo et al., 2019; Sivamaruthi et al., 2020).

## Periodontal Diseases

Periodontal diseases are chronic inflammatory diseases that destroy bone and gum tissues that support the teeth, of which gingivitis and periodontitis are the most common types. Gingivitis is a mild form of periodontal disease, but the progression of untreated gingivitis can lead to more serious periodontitis by creating deep periodontal pockets that could cause teeth to loosen or lead to tooth loss, which has a marked impact on patients’ life. It is reported that as of 2019, there are 1.1 billion patients with severe periodontitis worldwide, and the prevalence of severe periodontitis has increased by 8.44% from 1990 to 2019 (Chen et al., 2021). Dental plaque, which is a microbial biofilm that forms on the teeth and gingiva, is thought to be the initial factor of periodontal diseases. The understanding of the pathogenicity of dental plaque biofilms has evolved over time, and several hypotheses were proposed in history, from the “Specific Plaque Hypothesis” (1976) (Loesche, 1976), the “Non-Specific Plaque Hypothesis” (1986) (Theilade, 1986), to the “Ecological Plaque Hypothesis” (1994) (Marsh, 1994). Modern periodontology, however, not only focuses on the pathogenicity of dental plaque biofilms but also emphasizes the interaction between oral microbes and the host. In recent years, the “Keystone-Pathogen Hypothesis” (KPH) (2012) and polymicrobial synergy and dysbiosis (PSD) model (2012) have attracted wide attention. The KPH (Hajishengallis et al., 2012) proposed that certain low-abundance periodontopathogens such as *Porphyromonas gingivalis* could weaken the bactericidal effect of the host immune system and promote host inflammatory response, thus destroying the host–microbe homeostasis and balance of periodontal microecosystem that finally lead to the occurrence of periodontal diseases. The PSD model (Hajishengallis and Lamont, 2012) emphasized that the synergistic effect between polymicrobial communities and the host inflammatory response disorder caused periodontal diseases, and moreover, the ecological imbalance and inflammatory response could reinforce each other and constitute the actual driving factors of diseases. In fact, subversion of host immunity by dysbiotic periodontal microbiota not only gives rise to periodontal diseases but also contributes to systemic inflammation (Hajishengallis, 2015).

**Figure 1:**
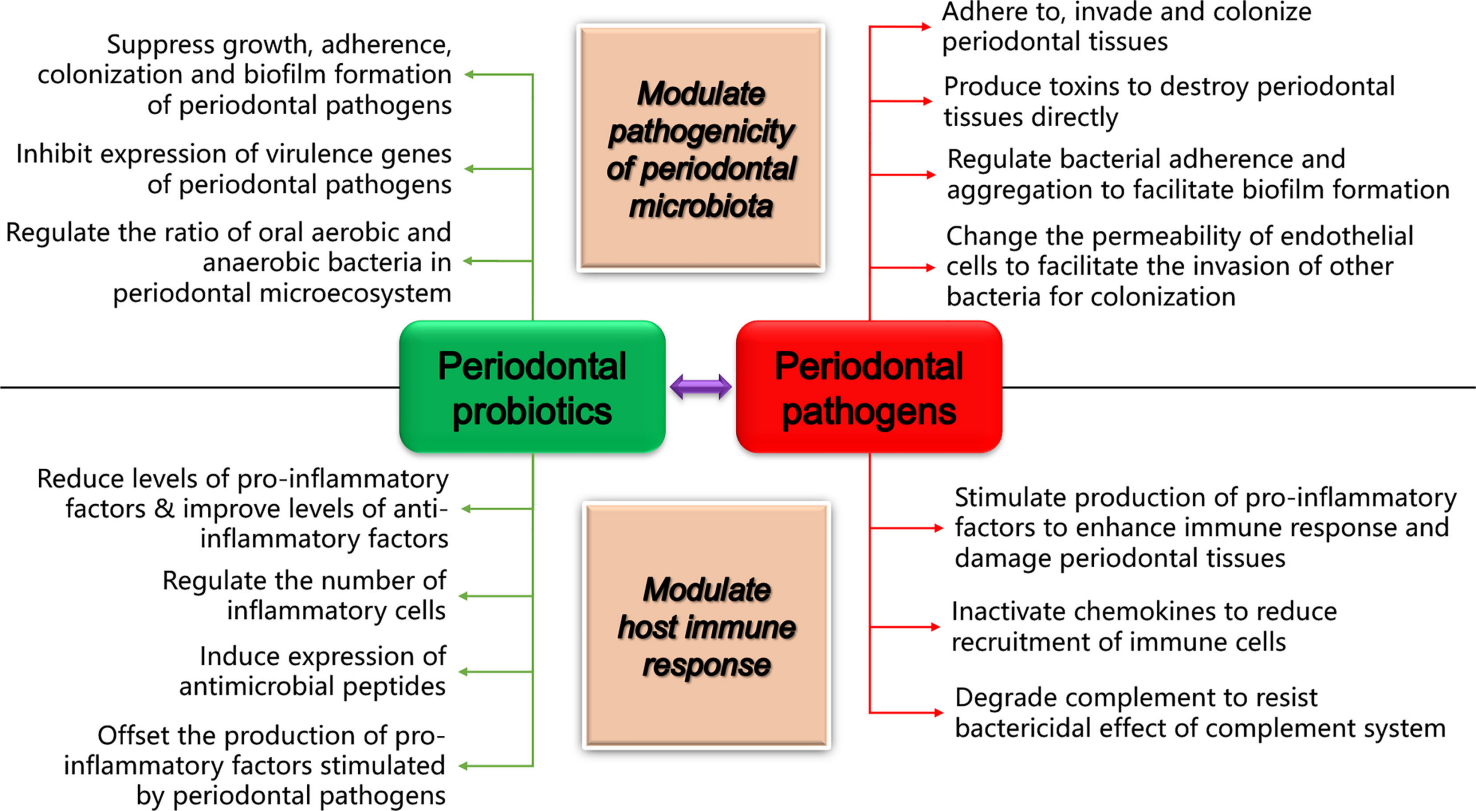
How periodontal probiotics and pathogens play their roles in regulating periodontal health and disease. The diagram shows the primary mechanisms of periodontopathogens and probiotics in regulating periodontal microbiota and host immune responses, respectively.

Studies on oral microorganisms show that there are more than 700 bacterial species colonizing the mouth (Kumar et al., 2005). However, only a few bacteria are proved to initiate and advance periodontal diseases, such as *P. gingivalis*, *Aggregatibacter actinomycetemcomitans*, *Tannerella forsythia*, *Prevotella intermedia*, and *Fusobacterium nucleatum* (Hajishengallis et al., 2012). When reviewing the studies focusing on the action of probiotics in managing periodontal diseases, we noticed that the majority referred to four periodontopathogens, namely, *P. gingivalis* (chronic periodontitis), *A. actinomycetemcomitans* (aggressive periodontitis), *P. intermedia* (pregnancy gingivitis, moderate and severe gingivitis, acute necrotizing gingivitis, and chronic periodontitis), and *F. nucleatum* (chronic periodontitis and acute necrotizing ulcerative gingivitis), indicating that periodontal probiotics are often related with or applied to specific periodontal diseases driven by them. With the help of various virulence factors that cause direct destruction to periodontal tissues or stimulate host cells to activate a wide range of inflammatory responses (**Figure 1**), these pathogens destroy the host–microbe homeostasis and cause or promote the occurrence and development of multiple periodontal diseases (Zhao et al., 2012; Jaffar et al., 2016; Zhao et al., 2019; Ishikawa et al., 2020; Moman et al., 2020; Ding et al., 2021; Jansen et al., 2021).

## Application of Probiotics in Managing Periodontal Diseases

There is an increasing interest in the use of probiotics in periodontal therapy and periodontal care. The existing published studies have revealed that probiotics could effectively inhibit periodontopathogens and improve various clinical indices related to periodontal health, including plaque index (PI), gingival index (GI), bleeding on probing (BOP), periodontal pocket depth (PPD), clinical attachment loss (CAL), and gingival crevicular fluid (GCF) volume, as well as inflammation-associated biochemical markers, such as interleukin (IL)-1β, matrix metalloproteinase (MMP)-8, and tissue inhibitor of metalloproteinase (TIMP)-1. Although there are various forms of probiotics applied in managing periodontal diseases, such as tablets, mouthwash, and toothpaste, probiotics commercialized and studied in periodontal therapy are usually made into tablets, while probiotics in the forms of mouthwash and toothpaste are often applied in periodontal health care.

### Periodontal Health Care

Some studies have focused on the role of probiotics in periodontal care. Amizic et al. found that probiotic toothpaste could prevent caries and periodontal diseases more effectively than non-probiotic toothpaste, and the capacity of the toothpaste to inhibit bacteria was even better than that of mouthwash. It was speculated that toothpaste could contact the tooth surface for a longer time, and it would be easy to enter gingival sulcus with the help of a toothbrush (Amizic et al., 2017). However, the study of Alkaya et al. (2016) had a different conclusion. Forty patients with gingivitis were recruited and divided into 2 groups, using placebo or experimental probiotic *B. subtilis*-, *Bacillus megaterium*-, and *Bacillus pumulus*-containing toothpaste, mouthwash, and toothbrush cleaner for 8 weeks. After evaluation of PI, GI, PPD, and BOP at baseline and 8 weeks, it was reported that there was no intergroup difference detected, suggesting that genetically distinct probiotics perhaps have different effects on periodontal health.

### An Adjuvant Therapy for Periodontal Non-Surgical Treatment

Some researchers have evaluated the short-term and long-term effects of probiotics as a supplementary therapy for periodontal non-surgical treatment. For example, in the study of Kuru et al. (2017), 51 periodontal healthy volunteers were first given non-surgical periodontal therapy, and 7 days later (baseline), they were randomized into two groups receiving yogurt containing either placebo or *Bifidobacterium animalis* subsp. *lactis* DN-173010 for 28 days, followed by a 5-day non-brushing period. PI, GI, BOP, PPD, GCF volume, and total amount and concentration of IL-1β in GCF were measured on days 0 (baseline), 28, and 33. It was reported that there was no intergroup difference detected on day 0 and day 28. However, after plaque accumulation, all parameters in the probiotic group were significantly better than those in the placebo group on day 33, indicating that the short-term use of probiotics has a positive effect on plaque accumulation and gingival inflammatory parameters, even without oral hygiene measures. The long-term clinical benefits of probiotic were proved by İnce et al. Thirty patients with chronic periodontitis were randomly given either *L. reuteri*-containing lozenge or placebo twice a day after scaling and root planing (SRP) for 3 weeks, and clinical parameters were collected at baseline and on days 21, 90, 180, and 360 after SRP. It was found that the probiotic group’s PI, GI, BOP, and PPD were much better than those of the placebo group at all time points. Decreased MMP-8 and increased TIMP-1 levels in GCF were found to be significant up to day 180. From day 90, mean values of attachment gain were significantly higher in the probiotic group (Ince et al., 2015). In a meta-analysis about the effects of using probiotics as the supplemental therapy after periodontal non-surgical treatment for 42–360 days, Kumar et al. concluded that probiotics could help reduce CAL significantly in moderately deep periodontal pockets. However, the three of four studies included for meta-analysis showed significant heterogeneity, though the risk of bias was low (Kumar and Madurantakam, 2017). Studies have suggested that short-term and long-term applications of probiotics can achieve certain clinical benefits, but relevant studies are not enough, and limited probiotics are involved.

As for peri-implant disease, some studies on the effect of *L. reuteri* ATCC PTA 5289 and DSM 17938 as adjuvant therapy to mechanical debridement revealed the potential of probiotics in treating peri-implant mucositis. Using *L. reuteri* for at least 30 days relieved PI, GI, PPD, and BOP in peri-implant mucositis and reduced the concentrations of IL-1β, IL-6, and IL-8 in GCF (Flichy-Fernández et al., 2015; Galofré et al., 2018). Moreover, compared to mechanical debridement followed by rinsing with 0.12% chlorhexidine (CHX), utilization of *L. reuteri* did not make a difference (Peña et al., 2019). However, with respect to implantitis, only one study reported significant PD and BOP improvement in 90 days after mechanical treatment and *L. reuteri* application (Galofré et al., 2018). *L. reuteri* might improve PI, but the subgingival microbial community of the implant was not changed markedly (Galofré et al., 2018; Tada et al., 2018; Laleman et al., 2020).

### Alternatives to Antimicrobials

Because of the overuse of antimicrobial drugs, antimicrobial resistance becomes serious day by day. Therefore, there is an urgent need to find alternatives to antimicrobials. In this background, Shah et al. compared the effects of probiotics and antibiotics as the supplemental therapies of periodontal non-surgical treatment (Shah and Gujjari, 2017). They recruited 18 patients with aggressive periodontitis and divided them into 3 groups after SRP, which were given *L. brevis* CD2, *L. brevis* CD2 with doxycycline, or doxycycline alone for 14 days. PI, GI, PPD, CAL, and salivary levels of *Lactobacillus* and *A. actinomycetemcomitans* were measured at baseline, 14 days, 2 months, and 5 months. It was reported that GI in all the three groups was significantly improved at 5 months, and the intergroup results were also statistically significant. Besides, *L. brevis* CD2 showed a similar effect to doxycycline, but a synergistic effect was not detected when the probiotic and doxycycline were given simultaneously. It was concluded that *L. brevis* CD2 could be used as an alternative to antibiotics to treat aggressive periodontitis, without the risk of promoting antibiotic resistance.

The broad-spectrum bactericide CHX is widely used in dental clinical therapy. In a study comparing effects of CHX, probiotics, and herbs, 45 healthy volunteers were randomly divided into three groups and used CHX mouthwash, probiotic mouthwash, or herbal mouthwash for 14 days. The probiotic mouthwash contained *Lactobacillus acidophilus*, *Lactobacillus rhamnosus*, *Bifidobacterium longum, Saccharomyces boulardii*; the herbal mouthwash contained Belleric Myrobalan (*Bibhitaki*), Betel (*Nagavalli*), and Meswak (*Salvadora Persica*). PI, GI, and Oral Hygiene Index-Simplified (OHI-S) were collected on days 0, 7, and 14. The effects of all three types of mouthwash were demonstrated to be similar, and there were few side effects reported, suggesting that both probiotic mouthwash and herbal mouthwash are effective substitutes for CHX mouthwash (Deshmukh et al., 2017).

## Periodontal Health-Promoting Probiotic Species and Their Regulatory Effects

The mechanisms of probiotics promoting periodontal health have not been fully elucidated. Nevertheless, a considerable amount of research results from clinical trials, animal experiments, and *in vitro* experiments have revealed that probiotics confer periodontal-health benefits upon the host by regulating periodontal microbiota or immune responses *via* various mechanisms (**Figure 1**). Specifically, regulatory effects of various periodontal health-promoting probiotics, which mainly belong to genera *Lactobacillus*, *Bifidobacterium*, *Streptococcus*, and *Weissella*, as well as the emerging recombinant probiotics, have been widely observed (**Table 1**). For the genera *Lactobacillus* and *Streptococcus*, probiotic species whose periodontal health-modulating effects were reported by at least three research articles in the past 5 years are chosen as the typical and important periodontal health-promoting probiotics in their genera to introduce here, as listed in **Table 1**.

**Table 1:** The regulatory effects of known probiotic species promoting periodontal health.

Genus/type	Species	Regulate immune responses	Regulate periodontal microbiota
***Lactobacillus***	*Lactobacillus acidophilus*	Reduce *Porphyromonas gingivalis*-induced pro-inflammatory IL-1β and IL-6/8 production in KB cells (*in vitro experiment*) (Zhao et al., 2012) Reduce the *Fusobacterium nucleatum*-induced pro-inflammatory IL-6/8 production in KB and HOK cells (*in vitro experiment*) (Kang et al., 2011; Ding et al., 2021) Antagonize the regulatory effect on the proliferation and apoptosis stimulated by *P. gingivalis* (*in vitro experiment*) (Zhao et al., 2019)	Inhibit the growth of *P. gingivalis* (*in vitro experiment*) (Zhao et al., 2011) Co-aggregate with *F. nucleatum* to interfere with adhesion and invasion (*in vitro experiment*) (Ding et al., 2021) Downregulate the virulence-associated factors (*mfa*1, *fim*A, *kgp*, *rgp*A, and *lux*S) of *P. gingivalis* (*in vitro experiment*) (Ishikawa et al., 2020) Downregulate the adhesion-associated factors (*fap*2) of *F. nucleatum* (*in vitro experiment*) (Ding et al., 2021) Downregulate the virulence-associated factors (*Ltx*A, *Cdt*B, *dsp*B, and *kat*A) of *Aggregatibacter actinomycetemcomitans* (*in vitro experiment*) (Ishikawa et al., 2021) Degrade *A. actinomycetemcomitans* biofilms by producing enzymes such as lipase (*in vitro experiment*) (Jaffar et al., 2016)
	*Lactobacillus brevis*	Produce arginine deiminase to reduce the level of pro-inflammatory factors (TNF-α, IL-1β, IL-6, and IL-17) (*animal experiment*) (Maekawa and Hajishengallis, 2014)	Promote a higher ratio between aerobic and anaerobic bacteria in ligature-associated microbiota (*animal experiment*) (Maekawa and Hajishengallis, 2014) Inhibit *A. actinomycetemcomitans* in saliva (*clinical trial*) (Shah and Gujjari, 2017) Inhibit the growth and biofilm formation of *Prevotella melaninogenica* (*in vitro experiment*) (Shah and Gujjari, 2017)
	*Lactobacillus casei*	Reduce the *F. nucleatum*-induced pro-inflammatory IL-6 production in oral epithelial cells (*in vitro experiment*) (Kang et al., 2011)	Reduce the abundance of *P. gingivalis*, *A. actinomycetemcomitans*, and *P. intermedia* in subgingival plaque (*clinical trial*) (Imran et al., 2015) Degrade *A. actinomycetemcomitans* biofilms by producing enzymes such as lipase (*in vitro experiment*) (Jaffar et al., 2016)
	*Lactobacillus fermentum*	Reduce the *F. nucleatum*-induced pro-inflammatory IL-6 production in oral epithelial cells (*in vitro experiment*) (Kang et al., 2011)	Degrade *A. actinomycetemcomitans* biofilms by producing enzymes such as lipase (*in vitro experiment*) (Jaffar et al., 2016) *Inhibit the growth of* P. gingivalis, P. intermedia, and A. actinomycetemcomitans (*in vitro experiment*) (Terai et al., 2015)
	*Lactobacillus gasseri*	Raise the level of mBD14 mRNA in gingiva, tongue, and saliva (*animal experiment*) (Kobayashi et al., 2017) Decrease the mRNA levels of IL-6 and TNF-α in gingiva infected by *P. gingivalis* (*animal experiment*) (Kobayashi et al., 2017)	Reduce the expression of *Ltx*A and *Cdt*B exotoxins by *A. actinomycetemcomitans* (*in vitro experiment*) (Nissen et al., 2014) Inhibit the growth of *P. gingivalis* and *P. intermedia* (*in vitro experiment*) (Terai et al., 2015) Decrease the colonization of *P. gingivalis* in gingiva (*animal experiment*) (Kobayashi et al., 2017)
	*Lactobacillus reuteri*	Reduce the *F. nucleatum*-induced pro-inflammatory cytokine IL-6 production in KB cells (*in vitro experiment*) (Kang et al., 2011) Raise the hemocyte density in *Galleria mellonella* infected by *P. gingivalis*, upregulating immune responses (*animal experiment*) (Geraldo et al., 2020; Santos et al., 2020) Reduce the level of MMP-8 and increase the level of TIMP-1 (*clinical trial*) (Ince et al., 2015) Inhibit the expression of pro-inflammatory factors (TNF-α, IL-1β, and IL-17) (*clinical trial*) (Szkaradkiewicz et al., 2014)	Inhibit the growth of *P. gingivalis*, *P. intermedia*, *A. actinomycetemcomitans*, and *F. nucleatum* depending on B12, presence of anaerobiosis, and substrate glycerol (*in vitro experiment*) (Geraldo et al., 2020; Santos et al., 2020; Jansen et al., 2021) Inhibit *P. gingivalis* in saliva, supragingival plaque and subgingival plaque, and *P. intermedia* in saliva (*clinical trial*) (Invernici et al., 2018) Reduce the load of *P. gingivalis* in peri-implant mucositis (*clinical trial*) (Galofré et al., 2018)
	*Lactobacillus rhamnosus*	Reduce the number of TRAP-positive cells and infiltrating inflammatory cells (*animal experiment*) (Gatej et al., 2018)	Inhibit the growth of *P. gingivalis*, *A. actinomycetemcomitans*, and *F. nucleatum* (*in vitro experiment*) (Moman et al., 2020) Reduce the biofilm biomass and viable counts in biofilm of *A. actinomycetemcomitans* by releasing postbiotics (*in vitro experiment*) (Ishikawa et al., 2021). Downregulate the virulence-associated factors (*Ltx*A, *Cdt*B, *dsp*B, and *kat*A) of *A. actinomycetemcomitans* (*in vitro experiment*) (Ishikawa et al., 2021)
	*Lactobacillus salivarius*	–	Inhibit *A. actinomycetemcomitans* in saliva and GCF (*clinical trial*) (Sajedinejad et al., 2017) Reduce the expression of *Ltx*A and *Cdt*B exotoxins by *A. actinomycetemcomitans* (*in vitro experiment*) (Nissen et al., 2014)
	*Lactobacillus johnsonii*, *Lactobacillus fructosum*, *Lactobacillus delbrueckii* subsp*. casei*	–	Degrade *A. actinomycetemcomitans* biofilms by producing enzymes such as lipase (*in vitro experiment*) (Jaffar et al., 2016)
***Bifidobacterium***	*Bifidobacterium animalis* subsp. *lactis*	Increase the expression of anti-inflammatory factors (IL-10, TGF-β1, OPG, and β-defensins) and reduce the expression of pro-inflammatory factors (TNF-α, IL-1β, IL-6, CINC, and RANKL) in gingival tissues of experimental periodontitis (*animal experiment*) (Oliveira et al., 2017; Ricoldi et al., 2017; Silva et al., 2021) Reduce the expression of IL-1β and the ratio of RANKL/OPG in gingival tissues of rats with periodontitis and metabolic syndrome (*animal experiment*) (Silva et al., 2021) Reduce IL-1β in GCF (*clinical trial*) (Kuru et al., 2017) Raise the expression of β-defensin, TLR4, and CD4 in gingiva (*clinical trial*) (Invernici et al., 2020)	Inhibit the growth of *P. gingivalis*, *P. intermedia*, *A. actinomycetemcomitans*, and *F. nucleatum* (*in vitro experiment*) (Invernici et al., 2020) Reduce the adhesion of *P. gingivalis* to buccal epithelial cells (*in vitro experiment*) (Invernici et al., 2020) Antagonize the biofilm formation of *F. nucleatum* and *P. gingivalis* (*in vitro experiment*) (Argandoña Valdez et al., 2021) Change the ratio between aerobic and anaerobic bacteria and the proportion of subgingival community in animal models (*animal experiment*) (Oliveira et al., 2017; Ricoldi et al., 2017) Reduce the level of *P. gingivalis*, *Treponema denticola*, *Fusobacterium nucleatum vincentii*, and *A. actinomycetemcomitans* in deep periodontal pockets, saliva, and dental plaque (*clinical trial*) (Alanzi et al., 2018; Invernici et al., 2018)
***Streptococcus***	*Streptococcus salivarius*	Inhibit the expression of IL-6 and IL-8 induced by *P. gingivalis*, *A. actinomycetemcomitans*, and *F. nucleatum* in gingival fibroblasts (*in vitro experiment*) (Adam et al., 2011; MacDonald et al., 2021).	Inhibit the growth of *P. gingivalis*, *P. intermedia*, *A. actinomycetemcomitans*, and *F. nucleatum* (*in vitro experiment*) (Moman et al., 2020; Jansen et al., 2021) Inhibit the adhesion of *A. actinomycetemcomitans*, *P. gingivalis*, and *P. intermedia* (*in vitro experiment*) (Sliepen et al., 2008; Van Hoogmoed et al., 2008; Sliepen et al., 2009)
	*Streptococcus dentisani*	Increase the secretion of IL-10 and decline the level of IFN-γ induced by *F. nucleatum* in HGF-1 (*in vitro experiment*) (Esteban-Fernandez et al., 2019)	Change cell wall structure of *P. intermedia* and induce cell lysis of *F. nucleatum* (*in vitro experiment*) (López-López et al., 2017) Suppress *F. nucleatum* and *P. gingivalis* growth and attachment to HGF-1 (*in vitro experiment*) (López-López et al., 2017)
	*Streptococcus cristatus*	Reduce the *F. nucleatum*-induced pro-inflammatory IL-8 production in oral epithelial cells (*in vitro experiment*) (Zhang et al., 2008)	Produce arginine deiminase ArcA to inhibit fimbrial gene (*fimA*) expression and biofilm formation of *P. gingivalis* (*in vitro experiment*) (Xie et al., 2000; Xie et al., 2007) Inhibit adhesion and colonization of *A. actinomycetemcomitans* (*in vitro experiment*) (Sliepen et al., 2008)
	*Streptococcus gordonii*, *Streptococcus sanguinis*, *Streptococcus mitis*	–	Reduce fimbrial gene (*mfa1*) expression of *P. gingivalis* (*in vitro experiment*) (Xie et al., 2000; Xie et al., 2007) Inhibit adhesion and colonization of *A. actinomycetemcomitans*, *P. gingivalis*, and *P. intermedia* on hard surfaces or epithelial cells (*in vitro experiment*) (Teughels et al., 2007; Sliepen et al., 2008; Van Hoogmoed et al., 2008)
***Weissella***	*Weissella cibaria*	Reduce the *F. nucleatum*-induced pro-inflammatory cytokine (IL-6 and IL-8) production in KB cells (*in vitro experiment*) (Kang et al., 2011) Inhibit NF-κB activation and NO production in response to periodontopathogen stimulation in macrophages (*in vitro experiment*) (Kim et al., 2020b) Reduce both the production of pro-inflammatory (TNF-α, IL-1β, IL-6) and anti-inflammatory (IL-10) cytokines (*animal experiment*) (Kim et al., 2020a)	Co-aggregate with F. nucleatum, T. denticola, and P. gingivalis and inhibit the growth of F. nucleatum and P. gingivalis (in vitro experiment) (Kang et al., 2006b; Jang et al., 2016) Interfere with the adhesion of F. nucleatum (in vitro experiment) (Kang et al., 2011) Produce acid, H_2_O_2_, and N-acetylmuramidase to inhibit F. nucleatum, P. gingivalis, and P. intermedia (in vitro experiment) (Lim et al., 2018) Reduce the amount of plaque and F. nucleatum, P. gingivalis, P. intermedia, and T. forsythia levels in the oral cavity and P. gingivalis level in gingival tissues (animal experiment) (Do et al., 2019; Kim et al., 2020a) Reduce F. nucleatum in GCF (clinical trial) (Kang et al., 2020).
**Recombinant probiotics**	Recombinant *Lactobacillus paracasei*	–	Express single-chain antibody fragments against RgpA gingipain to co-aggregate with *P. gingivalis* and kill it (*in vitro experiment*) (Marcotte et al., 2006)
	Recombinant *L. acidophilus*	Express FomA to induce the production of antibodies against FomA protein and prevent the infection of *F. nucleatum* and its co-aggregated *P. gingivalis* (*in vitro experiment*) (Ma et al., 2013)	Present similar antibacterial activity and antibiotic sensitivity to the wild *L. acidophilus*, and its adhesive ability was improved (*in vitro experiment*) (Ma et al., 2018)

GCF, gingival crevicular fluid; OPG, osteoprotegerin.

### Lactobacillus

*Lactobacillus* is a type of gram-positive facultative anaerobic or obligate anaerobic bacteria that widely colonize the human digestive system, urinary system, and reproductive system. Probiotics derived from the genus *Lactobacillus* have been used in the prevention and treatment of numerous gastrointestinal tract disorders, urogenital diseases, vaginal infection, atopic disease, food hypersensitivity, and oral diseases like dental caries, periodontal diseases, and oral candida infection (Lebeer et al., 2008; Ishikawa et al., 2015; James et al., 2016). The genus *Lactobacillus* contributes the majority of the current known periodontal health-promoting probiotic species.

#### Lactobacillus acidophilus

The studies of periodontal health-promoting *L. acidophilus* are concentrated on *in vitro* experiments. *L. acidophilus* plays an important role in the inhibition of *P. gingivalis* growth *in vitro* and regulation of the interaction between *P. gingivalis* and gingival epithelial cells (GEC) (Zhao et al., 2011; Zhao et al., 2012; Zhao et al., 2019). *L. acidophilus* ATCC 4356 could offset the pro-inflammatory process induced by *P. gingivalis* ATCC 33277 at both protein and mRNA levels and could antagonize the regulatory effects of *P. gingivalis* on the proliferation and apoptosis of GEC in a dose-dependent manner (Zhao et al., 2012; Zhao et al., 2019). Recently, *L acidophilus* LA5 was observed to downregulate multiple virulence factors of *P. gingivalis*, such as the fimbriae encoding genes *mfa*1 and *fim*A in *P. gingivalis* ATCC 33277 and *mfa*1 in *P. gingivalis* W83, the gingipains encoding genes *kgp* and *rgp*A, and the quorum-sensing gene *lux*S in ATCC 33277 or W83 interacting with GECs (Ishikawa et al., 2020).

*L. acidophilus* ATCC 4356 also shows its universal regulatory effects on different *F. nucleatum* strains, *via* inhibiting the expression of virulence factors and the adhesion ability of *F. nucleatum* or reducing the levels of cytokines in oral epithelial cells stimulated by *F. nucleatum*. It is worth noting that live and heat-killed *L. acidophilus* ATCC 4356 have similar adhesion ability to KB and HOK cells, and both forms do not affect the viability of cells (Ding et al., 2021). The live *L. acidophilus* ATCC 4356 has been proved to decrease the production of IL-6 in KB cells activated by *F. nucleatum* ATCC 10953 (Kang et al., 2011). In a recent study, the heat-killed *L. acidophilus* ATCC 4356 was observed to downregulate the expression of IL-6/8 in KB and HOK cells activated by *F. nucleatum* ATCC 23726, co-aggregate with *F. nucleatum* ATCC 23726 and ATCC 25586, and inhibit the expression of their virulence factor *fap*2 involved in adhesion and invasion, thus interfering *F. nucleatum* self-aggregation and adhesion to epithelial cells, which is another way to inhibit *F. nucleatum* infecting oral epithelial cells in addition to competing for adhesion sites (Ding et al., 2021). The heat-killed *L. acidophilus* ATCC 4356 drew interest, as it could avoid drug resistance and dysbacteriosis and could be more safe (Ding et al., 2021).

It was reported that live *L. acidophilus* JCM1021 could degrade biofilms of *A. actinomycetemcomitans* Y4 (more than 90%), and SUNY75 and OMZ 534 (more than 50%) by lipase and other hydrolases (Jaffar et al., 2016). Ishikawa et al. further revealed part of the mechanisms in *A. actinomycetemcomitans* (serotype b, JP2 clone) biofilm degradation with cell-free pH-neutralized supernatants (CFS) of *L. acidophilus* LA5 and NCFM (Ishikawa et al., 2021). *L. acidophilus* LA5 CFS could reduce the number of planktonic bacteria, as well as biofilm biomass and viable counts in biofilm by releasing postbiotics, which can assist antibiotics in removing *A. actinomycetemcomitans*, as bacteria in biofilm are difficult to eliminate. Besides, *L. acidophilus* LA5 CFS could downregulate the expression of vital virulence factors leukocyte toxin (*LtxA*) and cytolethal distending toxin (*Cdt*B) related to evading host defenses. Another strain, *L. acidophilus* NCFM CFS, could decrease biofilm biomass and viable counts in biofilm by postbiotics but downregulate the transcription of *dsp*B, hindering its application to control periodontitis. Moreover, both *L. acidophilus* LA5 and NCFM downregulated *kat*A, a gene encoding catalase, attenuating the resistance of oxidative stress of *A. actinomycetemcomitans*.

#### Lactobacillus reuteri

Many *in vitro* experiments proved the inhibitory effects of *L. reuteri* on periodontopathogens, which is probably attributed to its specific by-products, such as reuterin, which is a non-protein broad-spectrum antibiotic and could suppress the growth of many gram-positive/negative bacteria, yeast, and fungi (Stevens et al., 2011). *L. reuteri* ATCC PTA 5289 is a good inhibitor of many periodontopathogens, including *P. gingivalis* ATCC 33277, *P. intermedia* ATCC 25611, and *F. nucleatum* ATCC 25586, except for *A. actinomycetemcomitans* ATCC 33384 (Jansen et al., 2021). As for the forms of the probiotics, both live *L. reuteri* PTA 5289 and DSM 17938 and their CFS showed inhibition on *P. gingivalis* ATCC 33277 and *F. nucleatum* ATCC 25586, while only the live form of the two *L. reuteri* attenuated the growth of *A. actinomycetemcomitans* ATCC 29522 *in vitro* (Geraldo et al., 2020; Santos et al., 2020). Another subspecies, *L. reuteri* ATCC 55730, also inhibited the growth of *F. nucleatum* ATCC 10953, *P. gingivalis* ATCC 33277, and *A. actinomycetemcomitans* ATCC 33384 and protected HOK cells infected by periodontal pathogens from death (Moman et al., 2020). Besides, exopolysaccharide (EPS) produced by *L. reuteri* DSM 17938 benefits its adhesion to epithelial cells to compete with pathogenic bacteria for adhesion sites (Kšonžeková et al., 2016). In another *in vitro* experiment, *L. reuteri* KCTC 3594 was shown to inhibit the secretion of IL-6 induced by *F. nucleatum* in KB cells (Kang et al., 2011).

In clinical trials, application of *L. reuteri* inhibited *P. gingivalis* in saliva, supragingival plaque and subgingival plaque, and *P. intermedia* in saliva (Invernici et al., 2018). However, for peri-implant diseases, *L. reuteri* DSM 17938 and PTA 5289, could only reduce the load of *P. gingivalis* in patients with peri-implant mucositis (Galofré et al., 2018). In animal experiments, the live *L. reuteri* DSM 17938 and PTA 5289 could raise the hemocyte density in *Galleria mellonella* infected by *P. gingivalis* ATCC 33277, thereby upregulating immune responses (Geraldo et al., 2020; Santos et al., 2020). The clinical trials also proved the immunomodulatory effects of *L. reuteri* such as regulating the imbalance between MMP and TIMP (Ince et al., 2015) or reducing the production of pro-inflammatory cytokines (tumor necrosis factor-α (TNF-α), IL-1β, and IL-17) (Szkaradkiewicz et al., 2014), which could contribute to relieving inflammatory response and reducing periodontal tissues destruction.

#### Lactobacillus rhamnosus

*In vitro* experiments, *L. rhamnosus* could inhibit several vital periodontopathogens and downregulate the virulence factors about biofilm formation and immune escape in *A. actinomycetemcomitans* (Moman et al., 2020; Ishikawa et al., 2021). *L. rhamnosus* ATCC 53103 could inhibit the growth of *F. nucleatum* ATCC 10953, *P. gingivalis* ATCC 33277, and *A. actinomycetemcomitans* ATCC 33384 *in vitro* and could protect HOK cells infected by periodontal pathogens from death (Moman et al., 2020). *L. rhamnosus* Lr32 and HN001 CFS could reduce the biofilm biomass and viable counts in the biofilm of *A. actinomycetemcomitans* (serotype b, JP2 clone) by releasing postbiotics to facilitate antibiotics removing this pathogen (Ishikawa et al., 2021). Besides, *L. rhamnosus* Lr32 CFS could downregulate the expression of *Ltx*A and *Cdt*B to interfere with the process of evading host defenses. Both *L. rhamnosus* Lr32 and HN001 downregulated *kat*A, damaging the resistance of oxidative stress of *A. actinomycetemcomitans.* However, it was observed that *L. rhamnosus* HN001 CFS upregulated the transcription of *dsp*B to degrade EPS of the biofilm but raised the transcription of *Ltx*A, thus hindering its application to control periodontitis (Ishikawa et al., 2021). *L. rhamnosus* could also regulate *in vivo* immune responses. *L. rhamnosus* GG reduced inflammatory cell, osteoclast, and TRAP-positive cell number in periodontal tissues in a mouse model of experimental periodontitis (Gatej et al., 2018).

### Bifidobacterium

*Bifidobacterium* is a type of gram-positive anaerobic bacteria that could be found in the human intestines, vagina, oral cavity, and breast milk. *Bifidobacterium* probiotics have been proved to relieve multiple intestinal diseases such as irritable bowel syndrome and constipation (Agrawal et al., 2009) and inflammatory bowel disease (Kim et al., 2007), improve lactose intolerance (He et al., 2008), prevent infectious diarrhea (Qiao et al., 2002), and reduce incidence and duration of respiratory infections (Jungersen et al., 2014) and exhibit anticancer effects (You et al., 2004). They also play a role in controlling oral infectious diseases including periodontal diseases (Ricoldi et al., 2017) and oral candida infection (Ishikado et al., 2007).

#### *Bifidobacterium animalis* subsp. *lactis*

Recent studies have evaluated the effects of the *B. animalis* subsp. *lactis* (*B. lactis*) on periodontopathogens. In *in vitro* experiments, *B. lactis* ATCC 27673 antagonized the biofilm formation of *F. nucleatum* ATCC 25585 and *P. gingivalis* ATCC 33277 after co-incubating for 168 h, without interfering with the growth of *Streptococcus oralis* (Argandoña Valdez et al., 2021). *B. lactis* HN019 not only inhibits *P. gingivalis* W83, *P. intermedia* ATCC 25611, *F. nucleatum* ATCC 25586, and *A. actinomycetemcomitans* ATCC 33393 but also significantly reduces the adhesion of *P. gingivalis* W83 to buccal epithelial cells (Invernici et al., 2020). In animal experiments, *B. lactis* could also regulate the ratio between aerobic and anaerobic bacteria, as reported in the study of Ricoldi et al. (2017). Oliveira et al. had a consistent conclusion in their study since they found that the *B. lactis* HN019 treatment resulted in lower proportions of *P. intermedia*-like species in subgingival plaque of EP animals (Oliveira et al., 2017). In clinical trials, when *B. lactis* HN019 was taken for 30 days, *P. gingivalis*, *Treponema denticola*, and *F. nucleatum vincentii* were reduced markedly in deep periodontal pockets (≥7 mm) (Invernici et al., 2018). *B. lactis* BB-12 combined with *L. rhamnosus* GG could decrease *F. nucleatum* and *A. actinomycetemcomitans* in saliva and dental plaque as well as *P. gingivalis* in dental plaque, and the amounts of bacteria in saliva become lower (Alanzi et al., 2018).

*B. lactis* HN019 is observed to regulate immune responses in animal experiments and clinical trials. Oliveira et al. reported that the EP-*B. lactis* HN019 group presented higher levels of osteoprotegerin (OPG) and β-defensins as well as lower levels of IL-1β and receptor activator of nuclear factor-kappa B (NF-κB) ligand (RANKL) than the EP-only group (Oliveira et al., 2017). *B. lactis* HN019 application could markedly decrease the levels of IL-1β and the ratio of RANKL/OPG in rats with periodontitis and metabolic syndrome and could downregulate the expression of TNF-α and IL-6 in rats only with periodontitis (Silva et al., 2021). In clinical trials, when *B. lactis* HN019 was taken for 30 days, 4 weeks, or 15 days after SRP, the mean ratios between the levels of IL-1β or IL-6 and those at baseline in GCF were lower than those in groups without *B. lactis* HN019; fewer osteoclasts, increased expression of anti-inflammatory factors (IL-10 and TGF-β1), and reduced expression of IL-1β and cytokine-induced neutrophil chemoattractant (CINC) were also induced (Kuru et al., 2017; Ricoldi et al., 2017; Invernici et al., 2018). *B. lactis* HN019 treatment as adjuvant therapy of SRP for 30 days could obviously raise the expression of β-defensin-3, toll-like receptor 4 (TLR4), and cluster of differentiation (CD)-4 in gingiva (Invernici et al., 2020).

### Streptococcus

*Streptococcus* is a type of gram-positive, aerobic to facultatively anaerobic bacteria that is a member of the normal flora of the human mouth and intestines. Some *Streptococcus* spp. are identified as sources of invasive infections in humans that range from subacute to acute or even chronic, while others have been proved their health benefits in improving digestive problems such as ulcerative colitis (Ohland and Macnaughton, 2010) and antibiotic-associated diarrhea (Correa et al., 2005), regulating immunity (Dargahi et al., 2020) and treating various oral diseases including dental caries (Di Pierro et al., 2015), periodontal diseases (López-López et al., 2017; Esteban-Fernandez et al., 2019), oral candida infection (Ishijima et al., 2012) and halitosis (Jamali et al., 2016).

#### Streptococcus salivarius

The regulatory effects of *S. salivarius* on many periodontopathogens have been observed *in vitro* experiments. *S. salivarius* M18 shows stable inhibition to common periodontopathogens, including *P. gingivalis* ATCC 33277, *P. intermedia* ATCC 25611, *F. nucleatum* ATCC 25586, and *A. actinomycetemcomitans* ATCC 33384 (Jansen et al., 2021). Another strain *S. salivarius* K12 shows distinct inhibitory effects on *P. intermedia* ATCC 25611, *A. actinomycetemcomitans* ATCC 33384, *F. nucleatum* ATCC 10953, and *P. gingivalis* ATCC 33277 (Moman et al., 2020; Jansen et al., 2021). *S. salivarius* K12 could raise the viability of HOK cells infected by *P. gingivalis* and *F. nucleatum*, thus increasing the defense capability of epithelium (Moman et al., 2020). Apart from M18 and K12, *S. salivarius* TOVE could cause 1.5% and 71.3% reduction of *A. actinomycetemcomitans* adhesion by pre-colonization of glass coverslips (Sliepen et al., 2008) or epithelial cells (Sliepen et al., 2009), as well as inhibiting the adhesion of *P. gingivalis* and *P. intermedia* (Van Hoogmoed et al., 2008).

*S. salivarius* probiotic strains are also found to regulate immune responses *in vitro* experiments. *S. salivarius* K12 and M18 could inhibit immune activation by periodontopathogens and reduce the levels of IL-6/8 in human gingival fibroblasts stimulated by *A. actinomycetemcomitans*, *P. gingivalis*, and *F. nucleatum* when *S. salivarius* is co-incubated with pathogens and fibroblasts simultaneously or *S. salivarius* is pretreated with fibroblasts before infection (Adam et al., 2011; MacDonald et al., 2021).

### Weissella

*Weissella* is a type of gram-positive facultative anaerobes that are classified from the genus *Lactobacillus* and occur in a great variety of habitats, including human saliva, breast milk, intestines, feces, vagina, and skin. Among them, there are opportunistic pathogens as well as probiotic bacteria with beneficial effects such as antibacterial activities (Srionnual et al., 2007), antifungal activities (Quattrini et al., 2020), and immunoregulation (Lee et al., 2013). Certain probiotic *Weissella* such as *Weissella cibaria* strains have been shown to play a role in inhibiting dental caries, halitosis, and periodontal diseases (Kang et al., 2006a; Kang et al., 2006b; Jang et al., 2016; Do et al., 2019; Kim et al., 2020a).

#### Weissella cibaria

Some *W. cibaria* strains have shown strong antibacterial activities against periodontopathogens *in vitro* experiments. Kang et al. found that *W. cibaria* CMU, CMS2, and CMS3 co-aggregated most strongly with *F. nucleatum*, the proliferation of which was decreased by 5-log cycles as a result (Kang et al., 2006b). Jang et al. obtained a consistent result, as 95% *P. gingivalis* and *F. nucleatum* were inhibited because of co-aggregation with *W. cibaria* CMU (Jang et al., 2016). It can be inferred that co-aggregation with *W. cibaria* does not interfere with the colonization of periodontal pathogens but suppresses their growth. *W. cibaria* CFS was also found to be against pathogens, which is mainly related to acidic pH, the presence of hydroxyl, and the secretion of specific proteins with antimicrobial activities (Lim et al., 2018). Organic acids, including lactic acid, acetic acid, citric acid, fatty acids, and oleic acid, could interfere with the basic metabolism of the pathogens and inhibit the growth of *P. gingivalis* KCTC 5352, *F. nucleatum* KCTC 2488, and *P. intermedia* ATCC 25611. Besides, hydrogen peroxide (H_2_O_2_) in CFS could suppress *P. gingivalis* and *P. intermedia*, and *W. cibaria* CMU could produce the most H_2_O_2_ in commercial probiotics for oral healthcare (Lim et al., 2018). On the other hand, the bacteriocin-like compounds (BLCs) of CFS, *N*-acetylmuramidase, were only against the *P. gingivalis* effectively by binding to cell walls and causing lysis (Lim et al., 2018). In animal experiments, the application of *W. cibaria* significantly lowered the amount of plaque; the level of *F. nucleatum*, *P. gingivalis*, *P. intermedia*, and *T. forsythia* in the oral cavity; and the level of *P. gingivalis* in gingival tissues (Do et al., 2019; Kim et al., 2020a). A recent clinical trial indicated that taking *W. cibaria* CMU for 8 weeks significantly reduced *F. nucleatum* in GCF (Kang et al., 2020), corresponding to its strong co-aggregation ability with *F. nucleatum in vitro* (Kang et al., 2006b).

*W. cibaria* was also observed to regulate the inflammatory response *in vitro* experiments. *W. cibaria* CMU could reduce the production of IL-6 and IL-8 in KB cells activated by *F. nucleatum*, as well as the cell attachment of *F. nucleatum*, while the viability and co-aggregation of *W. cibaria* CMU may not play an essential role in this process (Kang et al., 2011). The anti-inflammatory activity of *W. cibaria* CMU is related to inhibiting NF-κB activation in response to periodontopathogen stimulation and NO production. In RAW 264.7 macrophages stimulated by formalin-inactivated *A. actinomycetemcomitans* ATCC 3338, *W. cibaria* CMU downregulated the expression of inducible NO synthase (iNOS) and the mRNA of IL-1β and IL-6 to reduce NO production. The inhibition of NF-κB inhibitor α (IκBα) kinase (IKK) phosphorylation, IκBα degradation, and the nuclear translocation of p65 were also observed in *W. cibaria* CMU-treated RAW 264.7 macrophages (Kim et al., 2020b). In animal models, *W. cibaria* CMU decreased the level of both pro-inflammatory (TNF-α, IL-1β, and IL-6) and anti-inflammatory (IL-10) cytokines (Kim et al., 2020a).

### Recombinant Probiotics

In addition to the application of conventional forms, such as the live, heat-killed, freeze-dried probiotics and the probiotics CFS, recombinant probiotics produced by genetic engineering could express more diverse antibacterial substances and present an enhanced antibacterial activity. A recombinant *Lactobacillus paracasei* strain was constructed by Marcotte et al. to express single-chain antibody fragments (scFv) against RgpA gingipain, a virulence factor of *P. gingivalis*, to co-aggregate with *P. gingivalis*; the antibacterial activity of *L. paracasei* was not damaged. It is worth mentioning that co-aggregation may facilitate the colonization of *P. gingivalis via L. paracasei* adhesion, but the pathogen may be killed by the locally high concentration of antibacterial substances secreted by *L. paracasei* (Marcotte et al., 2006). In 2013, Ma et al. reported a recombinant *L. acidophilus*, some wild strains of which have shown periodontal beneficial characteristics, expressing *F. nucleatum* outer membrane protein FomA. The recombinant *L. acidophilus* strain could stimulate the antibodies against FomA protein to prevent the infection of *F. nucleatum* and its co-aggregated pathogens such as *P. gingivalis* in periodontal tissues (Ma et al., 2013). Animal studies proved that oral administration of the recombinant *L. acidophilus* reduced the infection by *F. nucleatum* and *P. gingivalis* (Ma et al., 2013). Particularly, the recombinant *L. acidophilus* presents a similar antibacterial activity and antibiotic sensitivity to the wild *L. acidophilus*, and its adhesive ability is further improved (Ma et al., 2018). In these studies, the genome of parent probiotics is modified by genetic engineering techniques to construct recombinant strains of probiotic with new genetic characteristics, providing a new idea for the development and application of probiotics.

## Issues in Current Application of Periodontal Health-Related Probiotics

### Effectiveness

Although a multitude of studies have suggested that probiotics are beneficial to periodontal health, the effectiveness of probiotics in managing periodontal disease and health is still controversial. Even in studies that used the same probiotic bacteria, the observed improvements in clinical measurements, inflammation, and microbiota related to periodontal diseases are not consistent. The causes of the conflicting observations and conclusions on the effectiveness of probiotics in different periodontal-health research are complicated and diverse.

Multiple factors are considered to affect the results of probiotic research, such as probiotic species or strains, administration dosage or modes, sample size, the combination of different probiotics, and reaction time. In a randomized controlled clinical trial of Laleman et al. (2015), 48 periodontitis patients were included, divided evenly into two groups after SRP (baseline), and then given either a placebo or a probiotic tablet containing *S. oralis* KJ3, *Streptococcus uberis* KJ2, and *Streptococcus rattus* JH145 twice a day for 12 weeks. No significant difference in clinical indices including PPD, BOP, and CAL could be detected between the probiotic group and the control group at the baseline, 12-week, or 24-week time points. Nevertheless, a *post hoc* power analysis conducted by them revealed that eight times more patients were needed to show a statistically significant intergroup difference for PPD at 12 weeks. The results of the study indicate that sample size is a non-negligible factor that affects the observations of probiotic studies. In addition, for different probiotic strains, the effective dose and the best application mode should be considered first when studying the effects of probiotics in modulating periodontal health. Inappropriate dose or mode in applying probiotics may result in the failure in obtaining expected outcomes and correct conclusions. However, the problem is that up to now, there are not enough references to determine effective dose and application mode for numerous probiotic strains. As for the combination of probiotics, *L. reuteri* DSM 17938 could regulate *L. reuteri* ATCC PTA 5289 to stabilize its antibacterial activity (Jansen et al., 2021). The antibacterial activity of *P. gingivalis* is related to the double or triple combination of *B. longum*, *B. lactis*, and *Bifidobacterium infantis*. The growth of *P. gingivalis* was inhibited by 41.8% with exposure to *B. longum* with *B. lactis* and 50.1% to triple combination (Argandoña Valdez et al., 2021). Besides, reaction time is another factor, as 11.3% *F. nucleatum* was shown to be suppressed at 24 h after exposure to probiotics, and growth inhibition rates rose to 18.4%–51.6% until 72 h (Argandoña Valdez et al., 2021). Thus, the combination manner of probiotics and action time also affect the effectiveness of judgment. Consequently, sometimes, it is difficult for researchers to scientifically assess the effectiveness of probiotics.

Experimental design and evaluation indices selected of a study also have an important impact on the understanding of probiotic effectiveness. For example, in some studies, it is concluded that probiotic treatment was not able to significantly improve the clinical symptoms of periodontal diseases, which may be partly attributed to the inappropriate selection of evaluation indices. As observed by Montero et al. in a study evaluating the efficacy of the adjunctive use of probiotics on gingivitis (Montero et al., 2017), gingivitis subjects were recruited and administered with tablets containing placebo or the probiotic combination of *Lactobacillus plantarum*, *L. brevis*, and *Pediococcus acidilactici* for 6 weeks. Their results showed no significant differences in the average GI between the placebo and probiotic groups. When focusing on the change in the number of sites with higher GI scores (GI = 3 at baseline), a significantly higher reduction was observed in the probiotic group. In other words, the average GI may be not the optimum evaluation index for probiotic intervention in the study, because the improvement effect of probiotics on sites with severe inflammation was diluted by other sites when calculating the mean value of GI.

Sometimes, it is reported that although the clinical parameters are improved by probiotics, periodontal microbiota and related inflammatory factors do not show detectable differences. Keller et al. found that after treating moderate-gingivitis patients with tablets containing a mix of *L. rhamnosus* PB01, DSM 14869 and *Lactobacillus curvatus* EB10, and DSM 32307 for 4 weeks, BOP and GCF volume were obviously improved, while all the selected cytokines (IL-1β, IL-6, IL-8, IL-10, and TNF-α) in GCF and the salivary microbiome were unaffected by the intervention (Keller et al., 2017). The researchers speculated that cytokine concentrations in GCF of some samples were lower than the detectable levels and thus could not be analyzed and evaluated. In addition, subgingival plaques were not collected in the study for microbial diversity and abundance analysis, which may be one of the reasons for not observing microbial alterations.

What is particularly noteworthy is that a recent study on intestinal colonization by probiotics suggested that the effectiveness of probiotics might vary from person to person. Through using endoscopy to collect flora at multiple intestine sites to analyze the flora composition of volunteers who were given probiotic supplements, Zmora et al. found that humans featured person-, region-, and strain-specific mucosal colonization patterns of probiotics, hallmarked by predictive pretreatment microbiome and host features (Zmora et al., 2018). Consequently, probiotic interventions are likely to exert differential influences on different individuals, which is thought to possibly explain the high variability in probiotic effects on the host or gut microbiome observed in different studies. Since periodontal diseases are related to original periodontal microbiota and host immunity, differential colonization resistance and responsiveness of individuals to probiotics may also exist and impact the research results observed with probiotic use in periodontal diseases.

### Safety

With the deepening of probiotic research and the recognition of more potential probiotic species and strains, there has been growing concern about the safety of probiotics, in particular, when applied to humans with the purpose of managing diseases and improving health. In fact, there are some studies reporting that probiotics have limited benefits to the body and may even be harmful to health (De Groote et al., 2005; Doron and Snydman, 2015). It has been recommended by the WHO/FAO working group to conduct a series of safety assessments of probiotics including antibiotic resistance, toxin production, potential hemolysis, metabolic activities, and side effects in humans and post-market surveillance of commercial consumers (Araya et al., 2002). Classical probiotics such as some *Lactobacillus* and *Bifidobacterium* species that have a long history of use in fermented foods or dairy products are generally recognized as safe (Kang et al., 2019). However, there are no adequate systematic safety studies, and indeed, complications of probiotic use occur sometimes. Some studies have revealed that treatment with probiotics caused bacteremia, including the most commonly used *Lactobacillus* spp. (De Groote et al., 2005). Despite no existing evidence of the occurrence of bacteremia induced by probiotic use in periodontal therapy or care, people with damaged periodontal tissues or tooth bleeding after SRP seem to be susceptible to bacterial invasion, and therefore such a possibility could not be ruled out.

The application of probiotics may have unexpected impacts on host immune responses and microecology. Recently, Suez et al. (2018) evaluating the probiotic impact on post-antibiotic reconstitution of the intestinal host–microbiome homeostasis showed that antibiotic treatment enhanced human gut mucosal colonization by probiotics, and more importantly, compared to spontaneous post-antibiotic recovery, probiotics significantly delayed rather than aided in gut microbiome and host transcriptome reconstitution. It was noticed that probiotic presence led to elevated transcription levels of certain inflammatory mediators and antimicrobial peptides, which may affect the restoration of the original intestinal flora. In many probiotic-periodontal disease studies, probiotics were attempted to be used as adjuvant therapy after non-surgical periodontal therapy. However, it is reminded by this study that probiotic use may influence or even interfere with periodontal microbiome recolonization and restoration of periodontal microecological balance after removal of dental plaques by periodontal non-surgical treatment.

Collectively, these findings suggest that studies on periodontal health-promoting probiotics need to focus more on the safety in use, although there are few relevant adverse events reported. Just as the effects of probiotics depend on strain traits, each probiotic strain would be anticipated to have a different safety profile. Thus, it is quite essential to verify the identities, phenotypic characteristics, and non-pathogenicity of different probiotics for safe use in humans (Kang et al., 2019). Furthermore, it is proposed that perhaps the safety of a commercially available probiotic product depends not only on the probiotic organism but also on the other constituents of the product, whether in food or medicinal formulation (Doron and Snydman, 2015). This highlights the importance of systematic and persistent assessment of probiotic products by researchers in the overall process of probiotic research, development, and application.

## Concluding Remarks and Future Perspectives

The emergence of probiotics provides more options for the prevention and treatment of periodontal diseases. Different from antibiotics and bactericides that are widely used in clinical practice, probiotics generally play a role in periodontal therapy and healthcare through regulating host immune function and restoring the balance of periodontal microecology. Consequently, probiotics have unique advantages and considerable potential in application to maintain periodontal health.

Different probiotic species exert their periodontal health-regulatory effects through diverse mechanisms such as competition for adhesion sites to epithelial cells, antagonism against growth, biofilm formation and virulence expression of periodontopathogens, and influence on host immune responses. Currently, the majority of known periodontal health-promoting probiotics is derived from the classical probiotic genera *Lactobacillus* and *Biﬁdobacterium* and seems to be more effective and safe when applied in human health management. Nevertheless, taking account of the controversy on effectiveness and concerns on safety, these probiotics, as well as probiotics derived from other genera or remolded by genetic engineering techniques, need more investigation to support their role in periodontal therapy and care.

The studies on probiotic use in the intestine have highlighted a need for developing personalized probiotic approaches according to the host individual’s flora and immune status so that probiotics could better colonize and play a more effective role in promoting health. In order to ensure universal and persistent efficacy, such a personalized strategy should also be considered and developed when applying probiotics in the prevention and treatment of periodontal diseases. Furthermore, a diverse combination of different probiotic species and probiotic strains is probably one of the major development directions for probiotic application, which may provide combinational or synergistic effects on regulating host microbiota and immunity, especially when considering the high complexity of subgingival plaques and the presence of various oral environmental stress factors. Genetic engineering would provide more ideas and possibilities for probiotic research and development. The probiotic function could be further enhanced and improved by directional genetic modifications on existing probiotics that strengthen or remold their immunoregulation capabilities, antimicrobial activities against periodontopathogens, adaptive capacities to the oral environment, etc.

## Author Contributions

YZ and QG conceptualized the review. YZ drafted the manuscript, and QG edited the manuscript, with YD providing critical revisions. All authors contributed significantly and read and approved the final manuscript.

## Funding

This work was supported by grants from the Youth Science Fund Project of the National Natural Science Foundation of China (No. 81500842) and the Science and Technology Department of Sichuan Province (No. 2021YJ0133).

## Conflict of Interest

The authors declare that the research was conducted in the absence of any commercial or financial relationships that could be construed as a potential conflict of interest.

## Publisher’s Note

All claims expressed in this article are solely those of the authors and do not necessarily represent those of their affiliated organizations, or those of the publisher, the editors and the reviewers. Any product that may be evaluated in this article, or claim that may be made by its manufacturer, is not guaranteed or endorsed by the publisher.
